# Supplementary material for: Nonspecific Inhibition of IL6 Family Cytokine Signalling by Soluble gp130
Source: Int J Mol Sci. 2024 Jan 23;25(3):1363. doi: 10.3390/ijms25031363 (PMC10855816; doi:10.3390/ijms25031363)
Supplement: Supplementary file 1 [file ijms-25-01363-s001.zip › ijms-2812415-supplementary-sgp130 supp.pdf]

Supplementary Materials

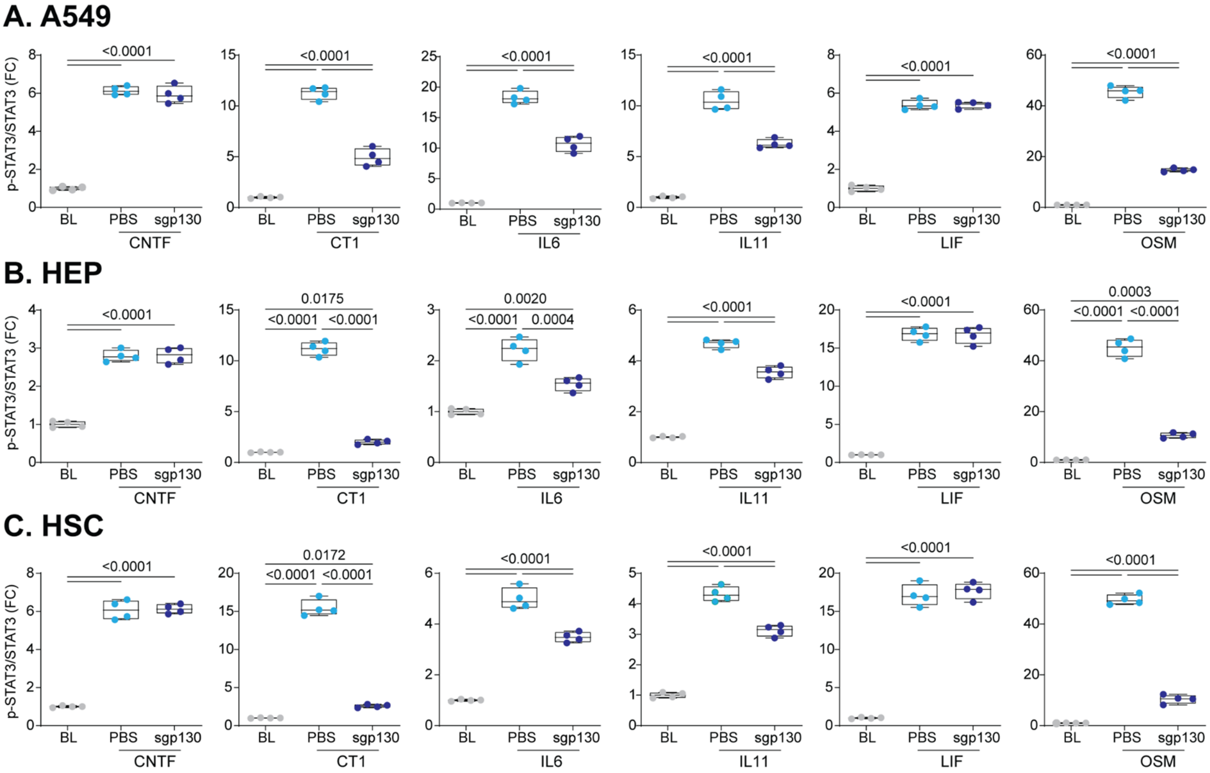

Supplementary Figure S1. Inhibition of STAT3 activation by sgp130Fc.

Densitometry analyses of p-STAT3 relative to total STAT3 expression in (**A**) A549, (**B**) HEP and (**C**) HCS following CNTF, CT1, IL6, IL11, LIF, or OSM (5 ng/mL) stimulation for 15 minutes in the presence of PBS or sgp130Fc (5 µg/ml). (**A-C**) Data are shown as box-and-whisker plots with median (middle line), 25th–75th percentiles (box) and min-max percentiles (whiskers); One-way ANOVA with Tukey's correction; n= 4 biological replicates. BL: baseline; FC: fold change.

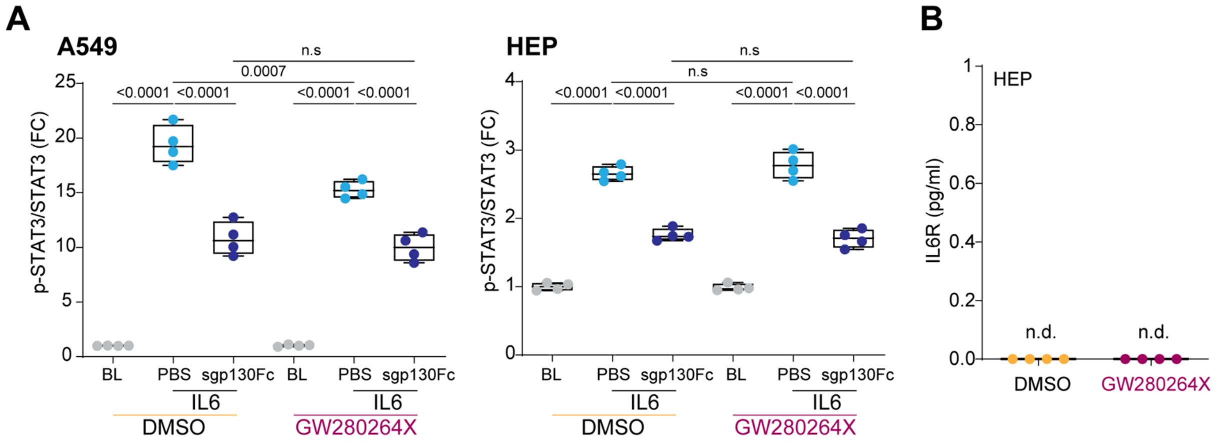

Supplementary Figure S2. Inhibition of STAT3 signalling by sgp130Fc in the absence of soluble IL6R.

(**A**) Western blots of STAT3 activation status in either DMSO or GW280264X-treated A549 (left) and HEP (right) in these following conditions: without stimulus (BL), IL6+PBS, or IL6+sgp130Fc. (**B**) ELISA of IL6R in the supernatant of HEP after 17 hours incubation with either DMSO or GW280264X. (**A-B**) Data are shown as box-and-whisker plots with median (middle line), 25th–75th percentiles (box) and min-max percentiles (whiskers); n = 4 biological replicates; DMSO (0.1%), GW280264X (1 µM), IL6 (5 ng/ml), sgp130Fc (5 µg/ml). (**A**) One-way ANOVA with Tukey's correction. BL: baseline; FC: fold change; n.d.: not detected.
