# Supplementary figures and images for: Nonspecific Inhibition of IL6 Family Cytokine Signalling by Soluble gp130
Source: Int J Mol Sci. 2024 Jan 23;25(3):1363. doi: 10.3390/ijms25031363 (PMC10855816; doi:10.3390/ijms25031363)

Uncropped blots

Figure 1B

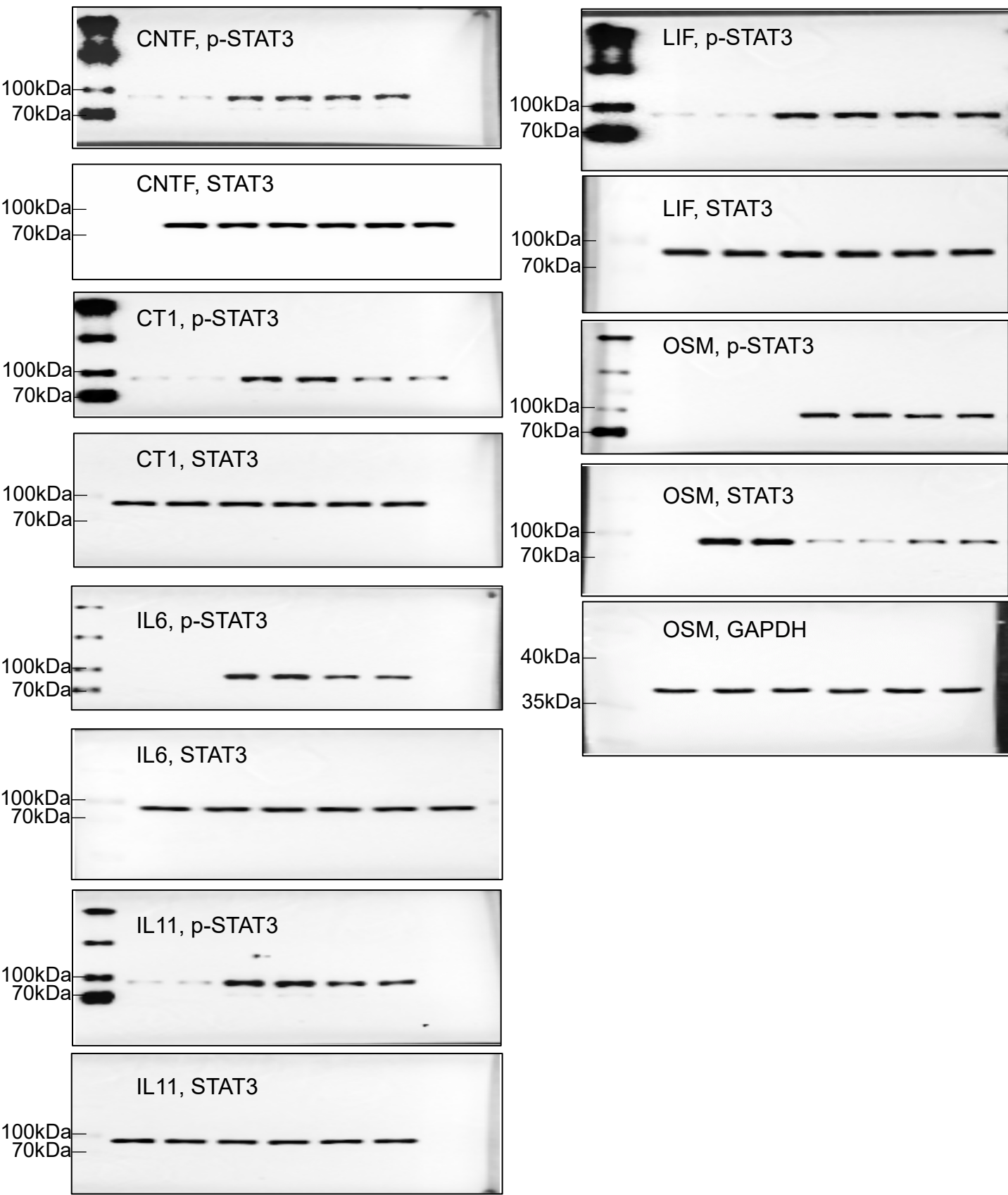

Figure 1C

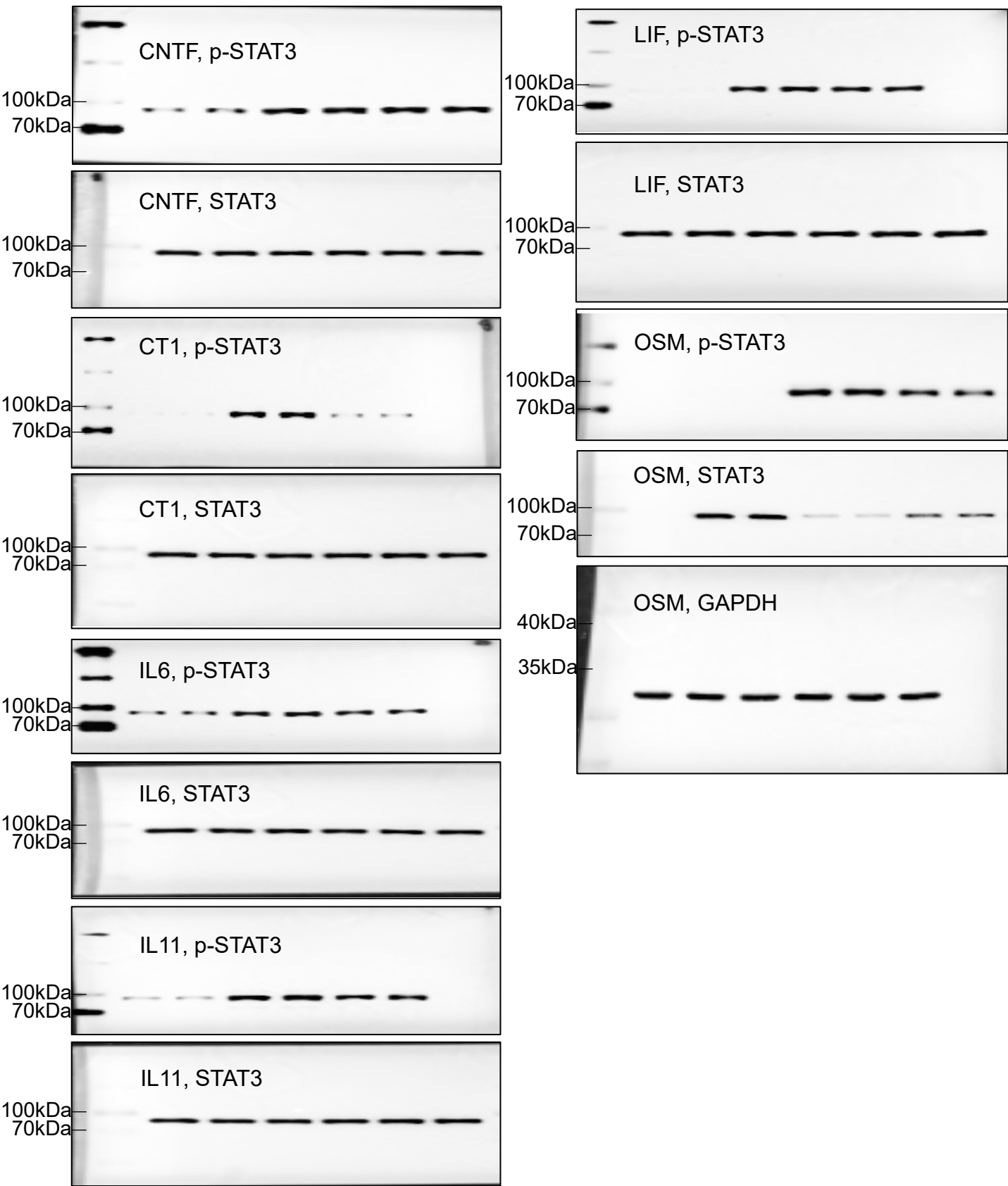

Figure 1D

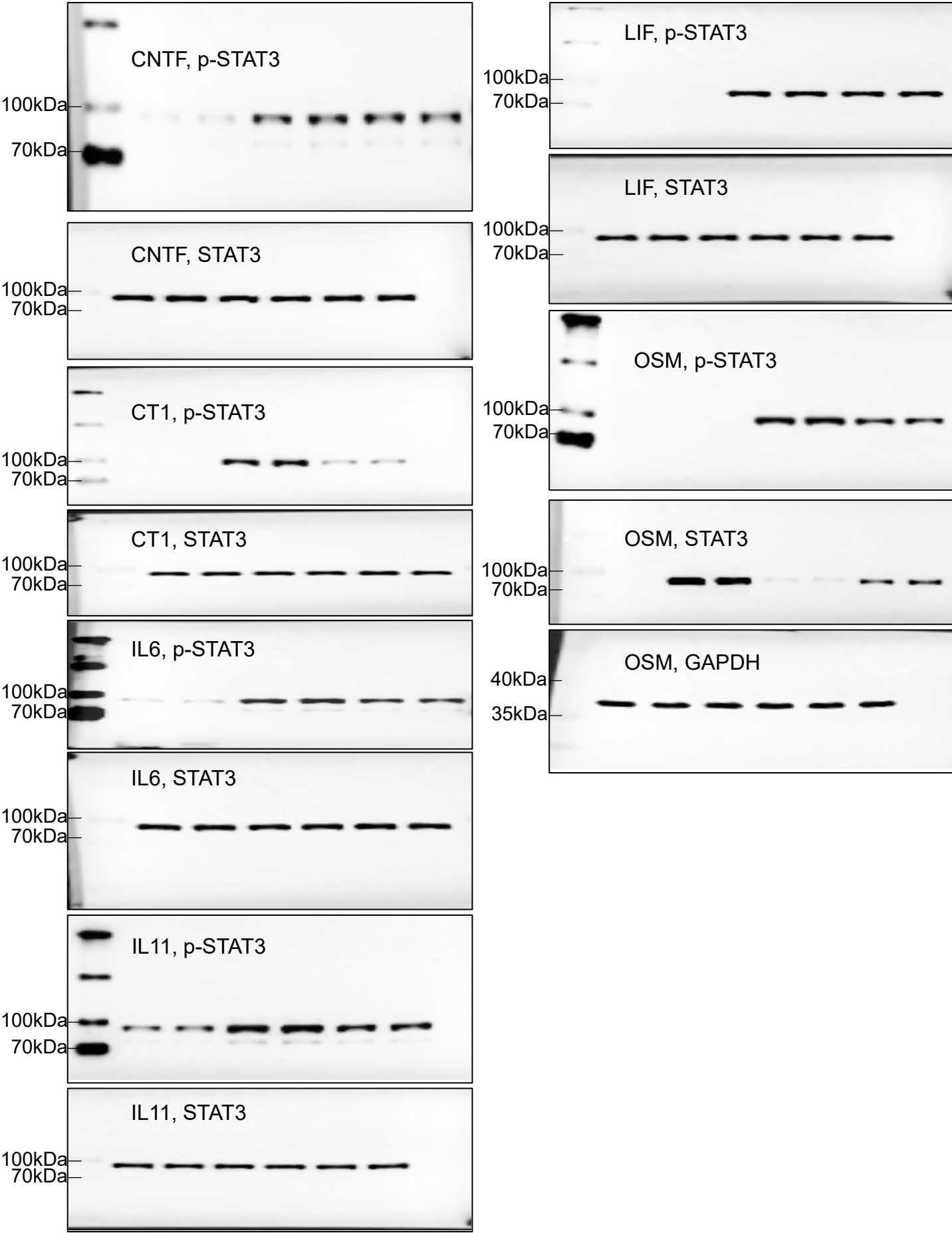

Figure 2A

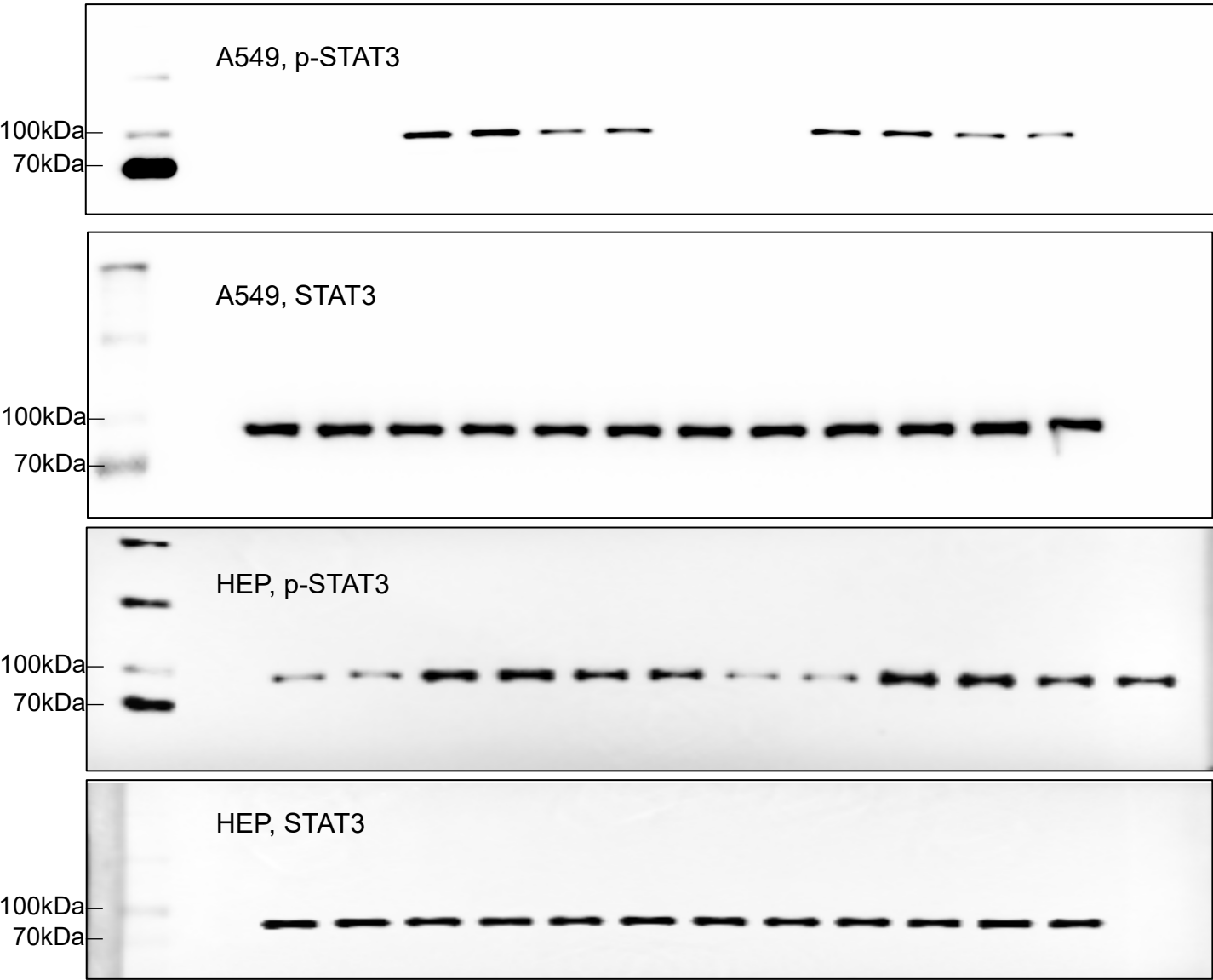

Figure 2C

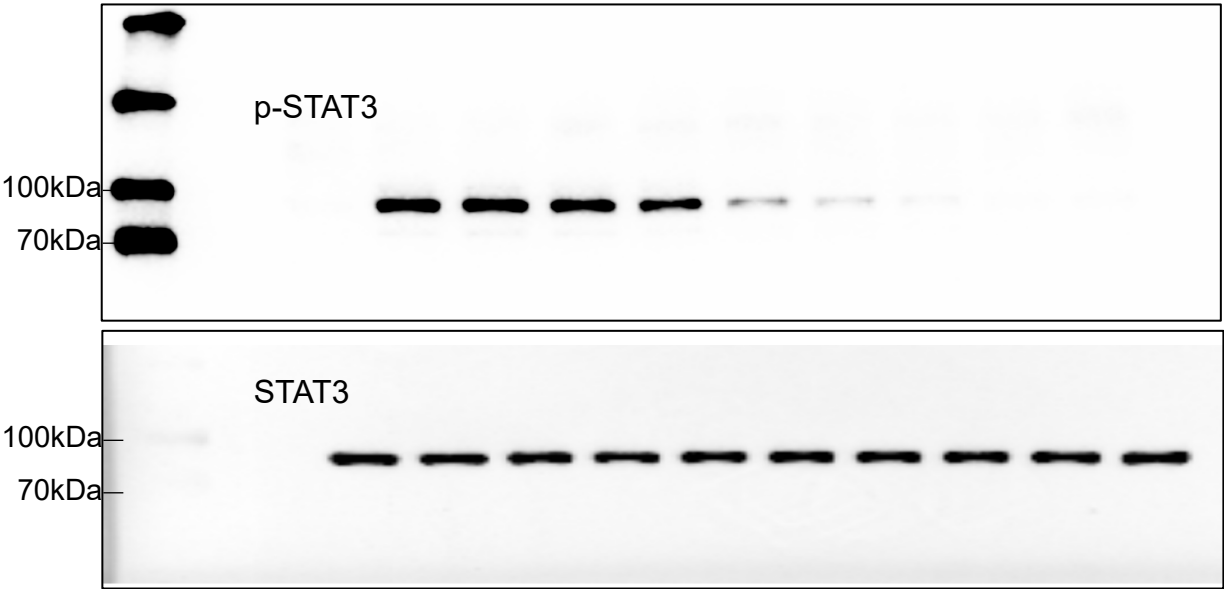

Figure 2E

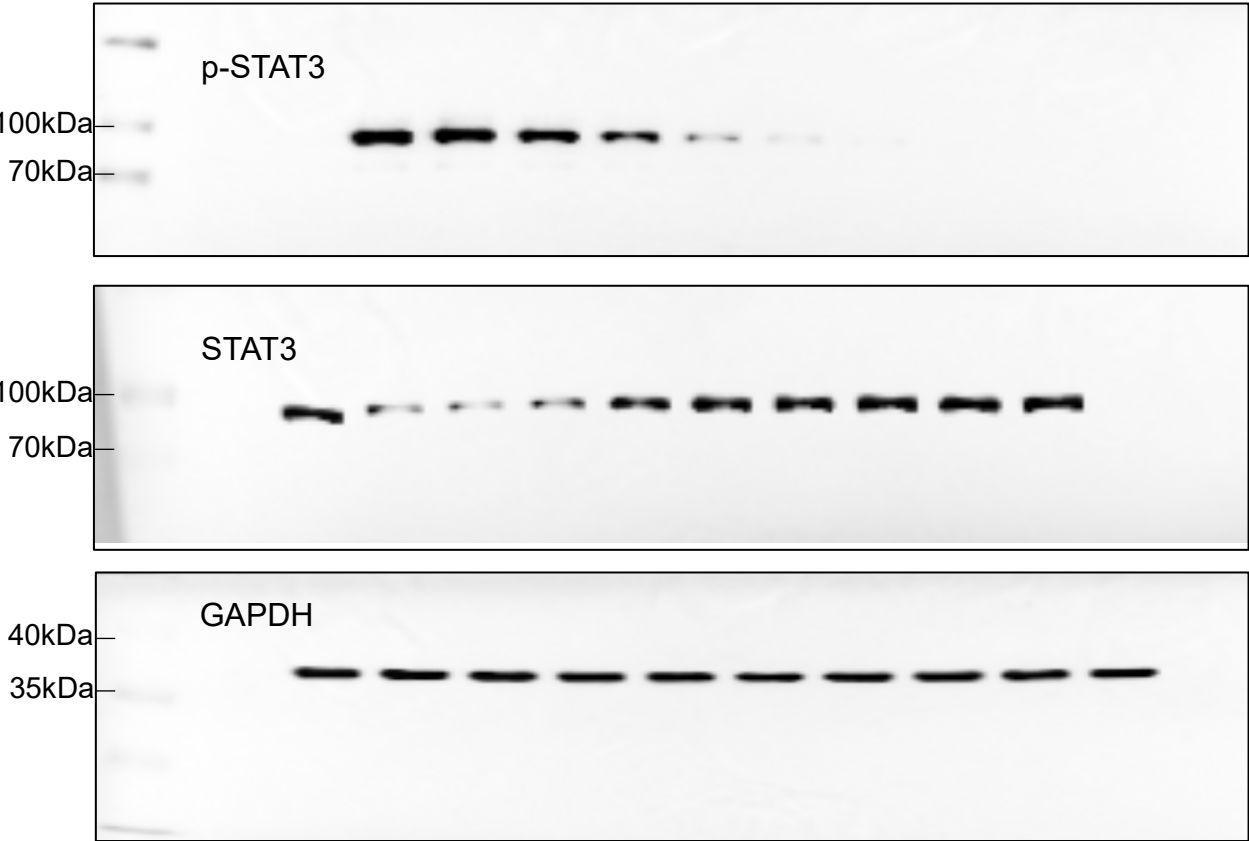

Figure 2G

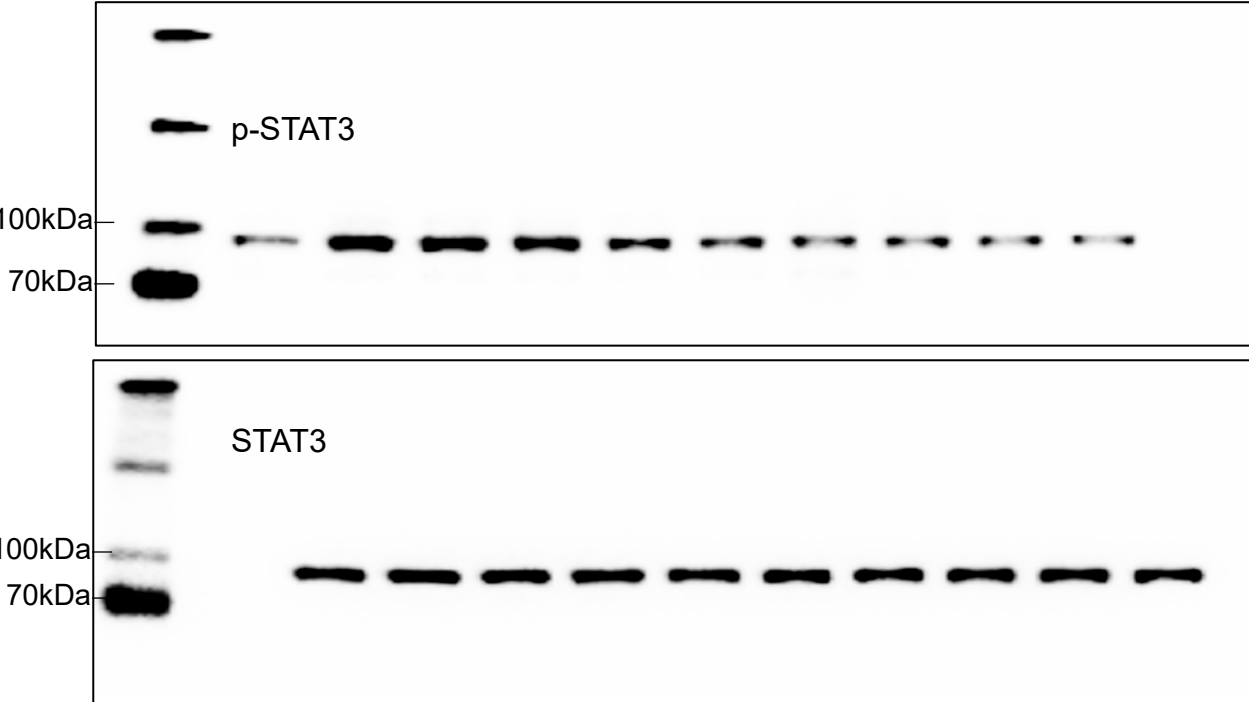

Figure 3A

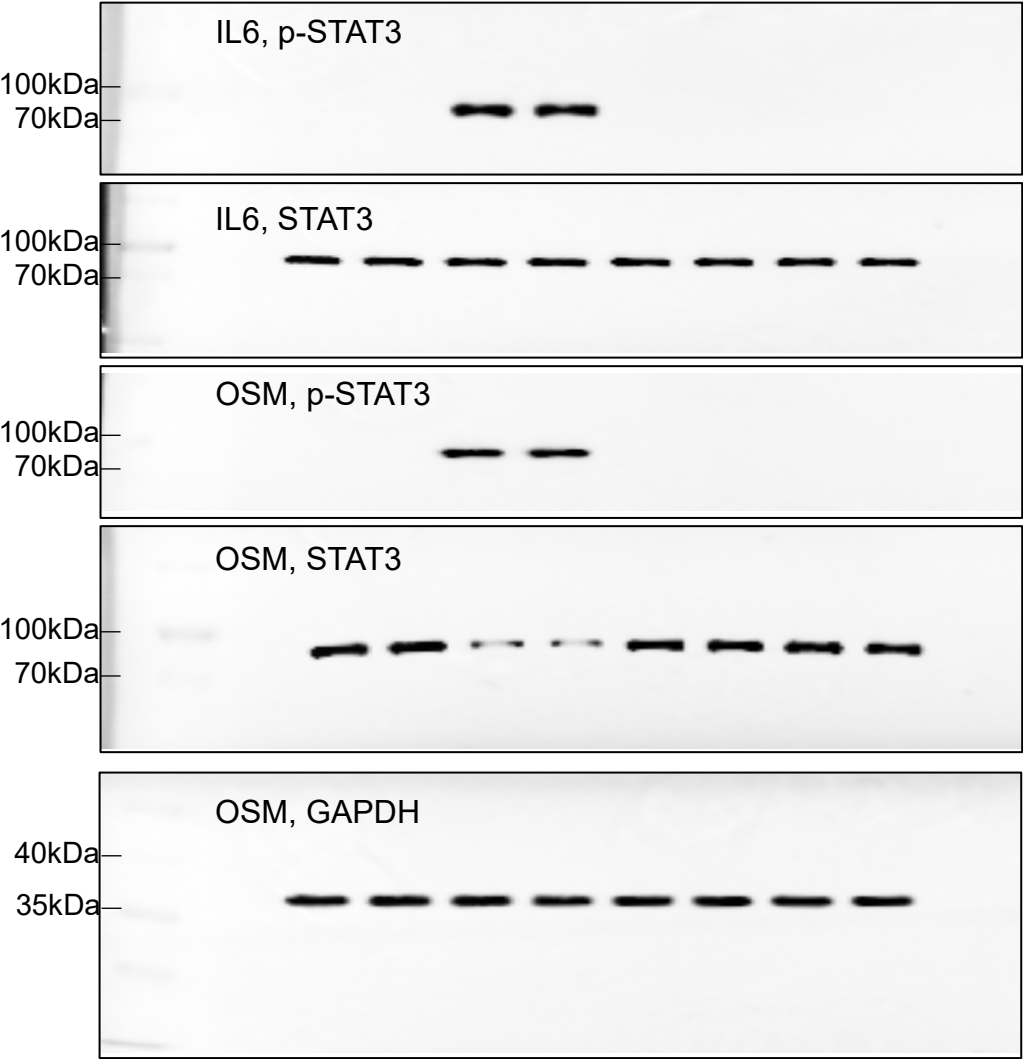

Supplement: Supplementary file 1 [file ijms-25-01363-s001.zip › ijms-2812415-supplementary-sgp130 uncropped blots.pdf]
